# Examination of the Impact of Strength and Velocity of the Knee and Ankle on Gait Speed in Community-Dwelling Older Adults

**DOI:** 10.3390/healthcare10102093

**Published:** 2022-10-20

**Authors:** Atsuki Kanayama, Mayuka Minami, Saki Yamamoto, Toshimitsu Ohmine, Minami Fujiwara, Takayuki Murakami, Shuji Okuno, Ryoga Ueba, Akira Iwata

**Affiliations:** 1 The Graduate School of Comprehensive Rehabilitation, Osaka Prefecture University, 3-7-30, Habikino 583-8555, Japan; 2 The Graduate School of Rehabilitation Science, Osaka Metropolitan University, 3-7-30, Habikino 583-8555, Japan; 3 Division of Physical Therapy, Department of Rehabilitation Sciences, Faculty of Allied Health Sciences, Kansai University of Welfare Sciences, 311-1, Kashiwara 582-0026, Japan

**Keywords:** plantarflexion, knee extension, angular velocity, muscle strength, mobility

## Abstract

The muscle strength of the knee extension and plantarflexion plays a crucial role in determining gait speed. Recent studies have shown that no-load angular velocity of the lower limb joints is essential for determining gait speed. However, no reports have compared the extent to which lower limb functions, such as knee extension strength, knee extension velocity, plantarflexion strength, and plantarflexion velocity, impact gait speed in a single study. Therefore, this study aimed to examine the relative importance of maximum strength and no-load angular velocity on gait speed. Overall, 164 community-dwelling older adults (72.9 ± 5.0 years) participated in this study. We measured the gait speed and lower limb function (the strength and velocity of knee extension and plantarflexion). Strength was measured with a hand-held dynamometer, and velocity with a gyroscope. A multiple regression analysis was performed with gait speed as the dependent variable and age, sex, and lower-limb function as independent variables. Plantarflexion velocity (*β* = 0.25) and plantarflexion strength (*β* = 0.21) were noted to be significant predictors of gait speed. These findings indicate that no-load plantarflexion velocity is more important than the strength of plantarflexion and knee extensions as a determinant of gait speed, suggesting that improvement in plantarflexion velocity may increase gait speed.

## 1. Introduction

Gait speed is a useful index for evaluating locomotion and is known to decline with age. The decline in gait speed can reach 12–16% per decade starting at the age of 60 years [1,2,3,4,5]. A slow gait speed is a good predictor of disability, hospitalization, falls, the requirement for a caregiver, and mortality in older adults in several epidemiological studies [1,2,3,4]. Gait speed is affected by various factors, such as health status, muscle function, sensory and perceptual functions, pain, motivation, cognitive status, and environment [6].

Lower limb muscle strength is a major determinant of gait speed [7,8,9,10,11]. Knee extension strength is strongly correlated with the gait speed of community-dwelling women, as reported in a study by Lord et al. [7]. Bendall et al. [9] reported that plantarflexion strength is related to gait speed in older adults. Furthermore, a decline in both knee extension and plantarflexion strength with aging is one of the factors that causes a decrease in gait speed [12]. Thus, knee extension and plantarflexion strength are critical determinants of the gait speed in older adults.

Lower limb muscle power is also involved in determining gait speed. Bean et al. [13] reported that muscle power is more closely associated with gait speed than muscle strength in older adults. Both velocity and strength are relevant determinants of muscle power, as muscle power is determined by the product of the two (power = velocity × strength). Therefore, the concept of angular velocity has gained attention. Recent studies in older adults have shown a relationship between gait speed and the angular velocity of the upper limbs [14,15], lower limbs [16,17,18], and trunk [19,20,21]. Regarding the lower limb, the angular velocity of knee extensions [16,17] and ankle plantarflexion [18] are correlated with the gait speed in older adults. Thus, the angular velocity of knee extension and ankle plantarflexion greatly contribute to gait speed.

As stated above, the knee and ankle joints significantly affect the gait speed of older adults, and strength and velocity play a particularly crucial role in determining gait speed. However, no previous reports have compared the extent to which knee extension strength, knee extension velocity, plantarflexion strength, and plantarflexion velocity contribute to gait speed in a single study. Thus, it remains unclear which is more important in determining gait speed: the knee or ankle, strength or velocity. This study aimed to comprehensively examine the importance of each of the four aforementioned lower limb functions in order to determine the gait speed in older adults. By clarifying the relative importance of lower-limb muscle strength and angular velocity in gait speed, this study could provide useful information regarding the effective interventions needed to improve gait speed in older adults.

## 2. Materials and Methods

### 2.1. Participants

In total, 164 community-dwelling older adults participated in this cross-sectional study. All participants were recruited through local newspaper advertisements and leaflets distributed at health-related events. The inclusion criteria were as follows: (1) age ≥ 65 years, (2) ability to walk independently without assistive devices, and (3) ability to understand and follow instructions. This study was registered in the University Hospital Medical Information Network Clinical Trials Registry (UMIN-CTR) under the number UMIN000041740. All procedures were performed in accordance with the ethical principles outlined in the Declaration of Helsinki, and the study was approved by the Human Ethics Committee of Osaka Prefecture University (approval number: 2019–118). Informed consent was obtained from all patients for study participation and the publication of information.

### 2.2. Measurements

We collected each participant’s demographic information (age, sex, height, and body weight), gait speed, and lower limb function (knee extension and ankle plantarflexion). All measurements were performed in a laboratory at the university by physical therapists in a random order on the same day. The gait speed was measured on an 8 m walkway using a photocell system (Optojump Next; Microgate, Bolzano, Italy). The initial and final 1.5 m sections were not timed to allow for acceleration and deceleration, and the time taken to walk 5 m in the center was measured [19]. Participants were instructed to walk as quickly as possible. After one trial session, the measurement was performed twice.

Measurements of the lower limb function were performed unilaterally on the right side, and the maximum value was used in the analysis for all measurements. The muscle strength and angular velocity of knee extensions were measured based on Van Roie’s method using an isokinetic dynamometer (Biodex system3; Biodex Medical Systems, Inc., Shirley, NY, USA) [16]. Participants were seated with their hips fixed at a 90° flexion, and their hips and shoulders were stabilized using safety belts. The rotational axis of the dynamometer was aligned with the transverse knee–joint axis and connected to the distal end of the tibia with a length-adjustable rigid lever arm. Knee extension muscle strength was evaluated by isokinetic movements at 60°/s in the knee flexion angle range of 90° to 20°. Participants were instructed to perform knee extensions with maximal effort while sitting on the isokinetic dynamometer. After one practice session, the measurement was performed twice. The strength data were normalized by body weight. Knee extension angular velocity was evaluated using a maximal unloaded knee extension test in the absence of any external resistance (except for the weight of the lever arm of the dynamometer) in a knee flexion angle range from 90° to 20°. Participants were instructed to perform knee extensions as quickly as possible while sitting on the isokinetic dynamometer. After two practice sessions, the measurement was performed in triplicate.

**Figure 1:**
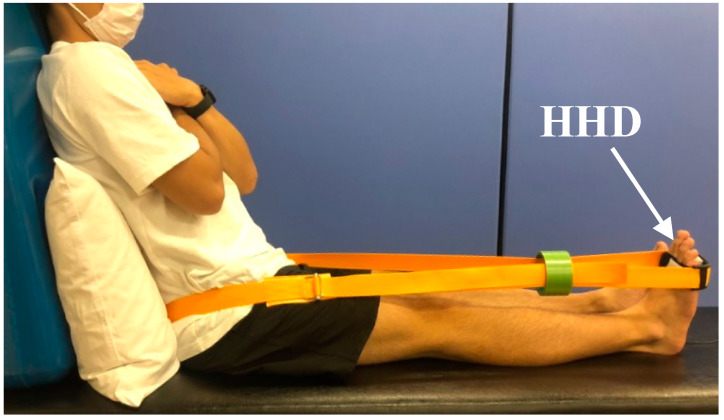
Measurement of ankle plantarflexion muscle strength. Participants sat on a bed with their backs against the backrest. The backrest was set to tilt backward by 30°. The hand-held dynamometer (HHD) was attached to the sole and the ankle joint was fixed to the neutral position.

Ankle plantarflexion muscle strength was measured using a handheld dynamometer (μTas-F100; ANIMA, Tokyo, Japan) (Figure 1) [22]. Participants were seated on a bed in a long sitting position, with their arms crossed in front of their chests. The sensor pad of the handheld dynamometer was placed at the distal end of the metatarsal bone at the sole of the foot. Thereafter, the ankle joint was fixed in a neutral position by tightening the belt. Participants were instructed to perform ankle plantarflexions with a maximum effort for 5 s. After one practice session, the measurement was performed twice. The strength data were normalized by body weight.

**Figure 2:**
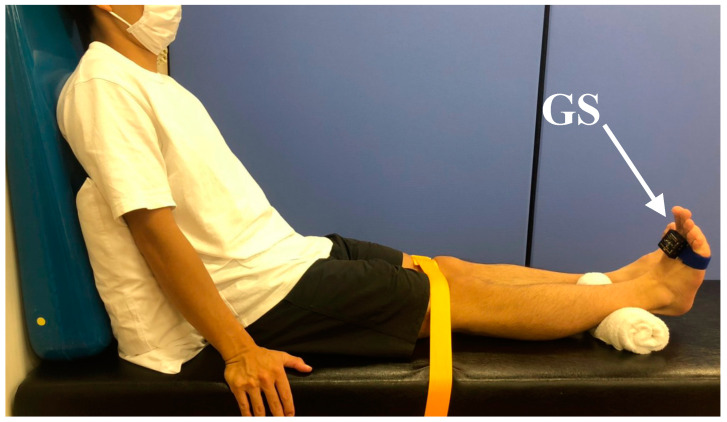
Measurement of ankle plantarflexion angular velocity. Participants sat on a bed with their backs against the backrest. The backrest tilted backward by 30°. The gyroscope (GS) was attached to the dorsum of the feet.

The ankle plantarflexion angular velocity was measured using a gyroscope (45 × 45 × 18 mm; MicroStone Corporation, Nagano, Japan) (Figure 2). The gyroscope was fixed onto the distal end of the second metatarsal bone at the dorsum of the foot so that the axis of the sensor was aligned with the sagittal plane [18]. The data from the gyroscope were captured at a frequency of 200 Hz. Participants were seated in a long sitting position on a bed with both hands on the floor. During the test, the lower limb was fixed to the bed using a belt at the knee. They were instructed to perform ankle plantarflexions as quickly as possible with ankle angles ranging from maximum dorsiflexion to maximum plantarflexion. The maximum velocity in the range of motion from maximum dorsiflexion to 50° was used in the analysis. After two practice sessions, the measurement was performed five times. The intra-tester reliability of the plantarflexion angular velocity was confirmed via preliminary experiments performed on 10 older adults (77.0 ± 9.0 years, Appendix A, Table A1). Plantarflexion velocity was measured twice after an approximately 1-week interval.

### 2.3. Statistical Analysis

In the preliminary study, the intra-tester reliability of the plantarflexion angular velocity using a gyroscope was used to assess the intraclass correlation coefficient (ICC 1.1).

Using G*Power 3.1, we calculated the sample size for multiple regression analysis with a medium effect size (f^2^) of 0.15; the number of predictors was six, with an α of 0.05, and a Power (1 − *β*) of 0.80 with reference to the method described by Cohen [23]. The minimum required sample size was 98. Statistical analyses were performed using the SPSS statistical software (SPSS version 25.0; IBM Corp., Armonk, NY, USA). The measurement data were presented as mean ± standard deviation and range (minimum-maximum). Pearson’s correlation coefficients were used to assess the relationship between gait speed and lower limb function (knee extension strength, knee extension velocity, plantarflexion strength, and plantarflexion velocity). Multiple linear regression analysis with forced entry was performed with gait speed as the dependent variable and age, sex, and limb function parameters as independent variables. We checked multicollinearity by variance inflation factors (VIF). If the VIF was 5 or larger, multicollinearity became a problem [24]. The statistical significance level was set at *p* < 0.05.

## 3. Results

In the preliminary study, the intraclass correlation coefficient for the test-retest reliability was 0.93 (*p* < 0.01), demonstrating the excellent reliability of the measurement (Appendix A, Table A2 and Table A3).

### 3.1. Basic Characteristics

**Figure 3:**
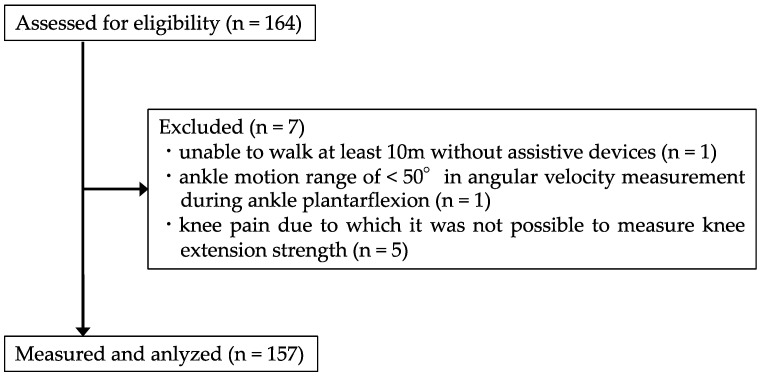
Flowchart depicting the selection of study participants.

Figure 3 shows a flowchart for the recruitment and exclusion of study participants. Seven of the initially selected 164 participants did not meet the inclusion criteria and were excluded from the study. Thus, 157 participants (41 men and 116 women) were included in the final analysis. Table 1 shows the characteristics of the participants. The mean age was 72.9 ± 5.0 years, height was 156.0 ± 8.0 cm, body weight was 54.8 ± 9.2 kg, and 23.1% of participants were male. Five of the 157 participants had a previous clinical history affecting the functional status of the lower limbs (two neurological diseases and three knee osteoarthritis).

### 3.2. Relationship between Gait Speed and Lower Limb Function

Table 2 shows Pearson’s correlation coefficients between the maximum gait speed and lower limb function. The maximum gait speed was significantly and positively correlated with all four lower limb functions (knee extension strength, r = 0.399, *p* < 0.01; knee extension velocity, r = 0.409, *p* < 0.01; ankle plantarflexion strength, r = 0.389, *p* < 0.01; ankle plantarflexion velocity, r = 0.394, *p* < 0.01).

Table 3 presents the results of the multiple linear regression analysis, which revealed that the independent variables accounted for 31% of the variance in the maximum gait speed (adjusted *R*^2^ = 0.31, *p* < 0.01). The ankle plantarflexion angular velocity (standardized *β*-regression coefficient = 0.25, *p* < 0.01), ankle plantarflexion muscle strength (standardized *β*-regression coefficient = 0.21, *p* < 0.01), age (standardized *β*-regression coefficient = −0.19, *p* < 0.01), and sex (standardized *β*-regression coefficient = −0.16, *p* < 0.05) were noted as significant predictors of the maximum gait speed. The VIF values for each predictor variable were acceptable, with all values under five.

## 4. Discussion

This is the first study to comprehensively examine the relationship between gait speed and lower limb function (the muscle strength and angular velocity of the knee and ankle) in older adults. The results of the study indicated two points: (1) the ankle function had a greater influence on gait speed than knee function, (2) the velocity was a more significant determinant of gait function than the strength, and (3) plantarflexion velocity was the most crucial factor for determining gait speed.

The first point can be explained by the differences in the roles of each joint during gait speed. Knee extension mainly provides shock absorption during the loading response and controls the body’s stability during mid-stance [25,26,27,28,29]. However, ankle plantarflexion provides a forward propulsion during push-off in the late stance [25,26,30,31], accounting for 67% of the total joint power during the stance phase of the gait [32]. The forward propulsion is a major factor in determining gait speed. The ankle function, which provides most of the propulsion, has a greater impact on gait speed than knee function.

In the second point, we consider that angular velocities in various regions have a significant impact on the mobility of older adults, as suggested by previous studies [16,17,18,33,34,35]. Sayers et al. [34] demonstrated that movement velocity in the leg press exercise is a stronger predictor of performance than muscle strength in lower-intensity tasks, such as gait. Furthermore, Yamamoto et al. [35] reported that knee extension velocity is more influential than knee extension strength as a determinant of gait speed in older adults. Our results that angular velocity is essential for gait speed are in line with the results of these studies.

We then discuss the third point, which is the main finding of this study. The swing speed during gait influences the control of gait speed in older adults [36]. Considering that the plantarflexion velocity during the push-off phase is one of the factors that determines the initial swing [37], this velocity can be regarded as a factor that affects gait speed. The plantarflexion angular velocity increases in the late stance and peaks during the late push-off phase (57–58% of the gait cycle) [38]. Most of the body weight is transferred to the contralateral leg during the late push-off phase [39]; therefore, the plantarflexion movement occurs in low-load conditions. In this study, we measured plantarflexion velocity without any load in the sitting position. Therefore, the load conditions were similar to the late push-off phase during gait and the velocity measurement in the present study. Therefore, this similarity may account for the finding that plantarflexion velocity in a sitting position is a crucial determinant of gait speed.

The plantarflexion angular velocity during heel-raising is the only method that has been shown to be related to gait speed [18]. Heel-raising is a high-load task for older adults [40]. Therefore, the angular velocity at a high-load task is greatly affected by the force component based on the force–velocity relationship [41]. Several studies [16,34] have suggested that lower limb angular velocity under low-load conditions is more strongly related to gait speed than under high-load conditions. We hypothesized that the plantarflexion velocity under low-load conditions is more important for gait speed, and therefore, this study measured angular velocity under no-load conditions. As a result, the no-load plantarflexion velocity had a particularly strong relationship with gait speed in older adults.

This study has several limitations. First, the method for measuring muscle strength differed between knee extension and ankle plantarflexion. In the measurement of knee extension strength, the measured value was calculated as torque (Nm/kg) using an isokinetic dynamometer. Contrarily, in the measurement of ankle plantarflexion strength, the measured value was calculated as force (kgf/kg) instead of torque using a handheld dynamometer. The differences in these measurement methods may have affected the results of this study. Second, we did not consider the dominant leg and measured only the right leg according to previous studies [11,16]. However, since large asymmetry (approximately 15%) has been observed in the strength of the lower limbs in older individuals [42], we should consider the dominant limb in future research. Third, a high percentage of participants were women (76.1%), and the age range was wide (65-89 years). Since previous studies have shown that gender and age affect the kinematics and kinetics of walking [43], these factors might influence the results of this study. We need to adjust for gender and age in future studies. Fourth, this examination included knee and ankle joints but did not evaluate the hip joint. The hip joint function might be related to gait speed. In addition, the subtalar joint kinematics affect the function of the ankle joint [44,45] but were not considered in this study. Therefore, further research should consider multiple joints, such as the hip and subtalar joints, and clarify the relationship between the gait and lower limb joint function in more detail. Finally, we did not consider the effect of the circadian rhythm on gait function, which was best in the evening and worst in the morning, in a published review [46]. As the data collection time points differed for the participants (between 9:00 am and 3:00 pm), the circadian rhythm may have influenced the gait-measurement results in this study.

## 5. Conclusions

The relative importance of gait speed in joints and functions is shown as follows: ankle > knee, and velocity > strength (in plantarflexion). Therefore, exercises that can increase plantarflexion velocity, such as ankle joint quick movements with unloaded or quick calf raises with weight-supports, may be effective for improving the gait speed of older adults whose gait function has declined.

## Figures and Tables

**Table 1:** Characteristics of participants (*n* = 157).

	Mean	±	SD	Range		
Age (years)	72.9	±	5.0	65	–	89
Sex (M/F)	41/116					
Height (cm)	156.0	±	8.0	140.5	–	176.0
Body weight (kg)	54.8	±	9.2	37.8	–	83.8
Maximum gait speed (m/s)	1.90	±	0.28	1.23	–	2.97
Knee extension strength (Nm/kg)	1.60	±	0.37	0.33	–	2.55
Knee extension velocity (°/s)	367.7	±	36.3	260.2	–	459.1
Ankle plantarflexion strength (kgf/kg)	0.86	±	0.27	0.22	–	1.72
Ankle plantarflexion velocity (°/s)	754.8	±	157.0	351.9	–	1261.0

SD: standard deviation, M: male, F: female.

**Table 2:** Correlation between gait speed and lower limb functions.

	Knee Strength	Knee Velocity	Ankle Strength	Ankle Velocity
Maximum gait speed (m/s)	0.399 **	0.409 **	0.389 **	0.394 **
Knee extension strength (Nm/kg)	−	0.644 **	0.534 **	0.221 **
Knee extension velocity (°/s)		−	0.386 **	0.278 **
Ankle plantarflexion strength (kgf/kg)			−	0.261 **
Ankle plantarflexion velocity (°/s)				−

**: *p* < 0.01.

**Table 3:** The results of multiple regression analysis.

	Maximum Gait Speed (m/s)		
Characteristic	Standardized *β*	*p*-Value	VIF
Age (years)	−0.19	0.01	1.2
Sex (M/F)	−0.16	0.04	1.3
Knee extension strength (Nm/kg)	0.10	0.28	2.1
Knee extension velocity (°/s)	0.11	0.23	1.9
Ankle plantarflexion strength (kgf/kg)	0.21	0.01	1.4
Ankle plantarflexion velocity (°/s)	0.25	<0.01	1.2
	Adjusted *R*^2^ = 0.31		

*β*: regression coefficient, VIF: variance inflation factor, M: male, F; female.

## Data Availability

The datasets generated or analyzed during the current study are available from the corresponding author upon reasonable request.

## Appendix A

**Table A1:** Characteristics of participants in the preliminary evaluation of the reliability of plantarflexion angular velocity (*n* = 10).

Characteristic	Mean	±	SD	Range		
Age (years)	77.0	±	9.0	60	–	90
Sex (M/F)	4/6					
Height (cm)	153.3	±	7.6	144.0	–	166.0
Body weight (kg)	57.3	±	12.1	41.2	–	71.0

SD: standard deviation, M: male, F: female.

**Table A2:** Ankle plantarflexion velocity in the preliminary evaluation.

Ankle Plantarflexion Velocity (°/s)	Mean	±	SD	Range		
Measurement 1	689.1	±	173.7	434.0	–	973.6
Measurement 2	680.7	±	215.2	393.0	–	1061.6

SD: standard deviation.

**Table A3:** The intraclass correlation coefficient for the test-retest reliability.

	ICC (1, 1)	95% CI			*p*-Value
Ankle plantarflexion velocity (°/s)	0.93	0.77	–	0.98	<0.01

ICC: intraclass correlation, 95% CI: the 95% confidence interval.
